# The Causes of Prolonged Infectivity in Scarlet Fever

**Published:** 1898-09-10

**Authors:** 


### THE CAUSES OF PROLONGED INFECTIVITY IN SCARLET FEVER.

The very important paper read by Dr. Kellick Millard on "The Etiology of 'Return Cases' of Scarlet Fever" shows one mode at least in which the infectivity of this disease may be retained in a patient, who may otherwise appear to have completely recovered from the attack. Out of 4,810 patients discharged from the Birmingham City Hospital, the return home was followed in 158 instances by a fresh outbreak of scarlet fever, at intervals varying from a few days up to six weeks, resulting in the admission to hospital of 171 so-called "return cases."

Dr. Millard protests against attempts being made to explain away such a sequence of events, and urges that we should rather endeavour to discover the conditions under which this recrudescence of the disease takes place. At the time of discharge a careful record was in all cases made of the exact condition of the patients as to desquamation, state of the fauces, and the existence of sores or other excoriations; of adenitis, skin eruptions, albuminuria, otorrhœa, and rhinitis. A comparison of the percentage of the various lesions in those cases that proved to be infecting, and in "all cases,"

showed that the incidence of these lesions was fairly uniform among the two classes, except in regard to rhinitis and otorrhœa. Thus, the returns relating to desquamation, adenitis, and abnormal throat showed that these conditions played little or no part in causing "return cases," their percentage of occurrence among infecting cases being even smaller than among "all cases." The case, however, was very different in regard to otorrhœa and rhinitis, both of which were in excess among the cases which proved to be infective. Especially was this the case with rhinitis, the very excessive proportion in which it occurred—nearly four to one—proving that this lesion, far more than any other, is a common precedent, or accompaniment of a prolonged infectivity. This is a matter which should receive the careful consideration of the profession. We have often protested against the common view which regards the skin and its desquamation as the main route by which infection escapes from the body. We will not enter further into this matter now, but it is evident that in regard to return cases some other cause must be sought for, and it would appear from Dr. Millard's statistics that the persistence of a discharge from the ear, and in a still greater degree the persistence of an unhealthy condition of the mucous membrane of the nose, is the active factor in causing certain cases of scarlet fever to remain infectious far beyond the normal time.

